# Supplementary material for: Gauss Markov and Flow Balanced Vector Radial Learning network traffic classification on IoT with SDN
Source: PLoS One. 2024 Oct 1;19(10):e0308052. doi: 10.1371/journal.pone.0308052 (PMC11444398; doi:10.1371/journal.pone.0308052)
Supplement: Attachment — Submitted filename: Reviewer Response sheet (1).docx [file pone.0308052.s001.docx]

**Review Comments to the Author**

Please use the space provided to explain your answers to the questions above. You may also include additional comments for the author, including concerns about dual publication, research ethics, or publication ethics. (Please upload your review as an attachment if it exceeds 20,000 characters)

**Reviewer #1:** **Correction is made in red color**

Before the Editor makes a decision, I suggest that the authors must take into account the following corrections:
1. I don't think the abbreviations used in the title are inspired.

**Response: [Correction is made in title]**

**Gauss Markov and Flow Balanced Vector Radial Learning Traffic Classification in Software-defined Networking**

2. I think the title needs to be reformulated to become more “friendly”.
In its current form, it doesn't really make meaning.

**Response: [Correction is made in title]**

**Gauss Markov and Flow Balanced Vector Radial Learning Traffic Classification in Software-defined Networking**

3. The "Introduction" section should be more concise. Also, Section 2.

**Response:**

**Correction is made in introduction and section 2.**

4. What is the motivation of the values in matrix (1)?

**Response: [Correction is made in below equation 2]**

The network traffic information matrix is used to arrange the data. The traffic matrix portrays how much traffic enters the site, its distribution inside the site, and at what places the traffic exits the site. This vehicle flow information is vital for precise network planning. A traffic matrix is important for network planning. Based on the matrix, the vehicle flow information is easily accessed to identify the traffic for reducing the complexity. The network traffic information is easily searched for discovering traffic.

5. Author must argue how the relation (2) was obtained, or indicate a bibliographic reference for its original form.

**Response: [**reference added in equation 2]

The Gauss Markov Worthiness is mathematically defined in [23] as a formula (Equation 2).

[23] Marc Hallin, (2014) “Gauss–Markov Theorem in Statictics”, Wiley StatsRef: Statistics Reference Online.

6. It is not clear how were obtained the relation (3).

**Response:**

The above equation 3 is elaborated in equation 4.

7. The right member of relations (3) and (4) is the same. Then why the need for Cor[A_{FF}]?

**Response:**

The above equation 3 is detailed elaborated in equation 4. From the above equation (4), the average feature class correlation ‘‘$Cor \left[ A_{FF} \right]$’’ is evaluated based on the sum of all the network features ‘$F_{1}+F_{2}+F_{3}+\ldots.+F_{n}$’ and the total number of network features ‘$n$’, respectively.

8. Details on obtaining relation (8) are required.

**Response: [Correction is made in below equation 8]**

From the above equation (8), Exponential kernel relationship between the two features is $K\left( E_{i}, E_{j} \right)$ measured based on mean and standard deviation in equation 9 and 10. ‘$E$’ denotes the sample extracted network feature vector, ‘$M$’ indicates the mean of the extracted network feature vector, and ‘$SD$’ represents the standard deviation of the extracted network feature vectors respectively.

9. Relation (11) is carelessly edited.

**Response: [Correction is made in below equation 11]**

$W\left( E \right)= \left\{ \begin{aligned} -1, Malicious route \\ &&&& \\ +1, Normal routing \end{aligned} \right.$	(11)

$W\left( E \right)= \left\{ \begin{aligned} if \left( tx_{bytes}<3500 \right)& and \left( dur<15 \right)and \left( pktcount<5000 \right), then malicious and discard route \\ &&&& \\ +1, then normal and optimal route \end{aligned} \right.$ (12)

From the above equations (11) and (12), with the above resultant weight values at the controller of the SDN, network traffic flow are classified as usual, following optimal routing. The optimal routing is denoted as ‘$+1$’. Or else, the network traffic flow is classified as malicious, following the discarding of the route. The malicious routing is represented as ‘$-1$’.Alternatively, network traffic flow is categorized as malicious after discarding the route.

10. Details on obtaining the data in Tables are required.

**Response: [**Tables are mentioned in section 4.2.2, 4.2.4 and 4.2.6]

**Table 4 Classification accuracy Comparison of Proposed GM-FVRL versus Existing IPro [1], MACCA2-RF&RF [2], and Novel hybrid DL model based on CNN [10]**

| Network traffic samples | Classification accuracy (%) | | | |
| --- | --- | --- | --- | --- |
|  | GM-FVRL | IPro | MACCA2-RF&RF | Novel hybrid DL model based on CNN |
| 500 | 98 | 97 | 94 | 97.25 |
| 1000 | 97.35 | 96.15 | 93.85 | 96.85 |
| 1500 | 97.15 | 95 | 93.15 | 95.85 |
| 2000 | 97 | 94.85 | 93 | 95.15 |
| 2500 | 96.85 | 94.25 | 92.85 | 94.75 |
| 3000 | 96.35 | 94 | 92 | 94.55 |
| 3500 | 96 | 93 | 91.85 | 93.85 |
| 4000 | 95.15 | 92.55 | 91 | 93 |
| 4500 | 94 | 92.15 | 90.85 | 92.85 |
| 5000 | 93.25 | 92 | 90 | 92.55 |

**Table 6 Recall Comparison of Proposed GM-FVRL versus Existing IPro [1], MACCA2-RF&RF [2], and Novel hybrid DL model based on CNN [10]**

| Network traffic samples | Recall (%) | | | |
| --- | --- | --- | --- | --- |
|  | GM-FVRL | IPro | MACCA2-RF&RF | Novel hybrid DL model based on CNN |
| 500 | 96.96 | 94.94 | 93.75 | 95.25 |
| 1000 | 95.15 | 91.25 | 90.25 | 93.55 |
| 1500 | 94 | 91.15 | 89.15 | 93 |
| 2000 | 93.25 | 90 | 88 | 91 |
| 2500 | 91 | 89.25 | 87.35 | 90.15 |
| 3000 | 91 | 88 | 86 | 89 |
| 3500 | 91.35 | 89.15 | 87 | 90.25 |
| 4000 | 92.55 | 89.35 | 88.25 | 91.55 |
| 4500 | 94.35 | 90 | 89 | 92 |
| 5000 | 95 | 91 | 90 | 93 |

**Table 8 F-measure Comparison of Proposed GM-FVRL versus Existing IPro [1], MACCA2-RF&RF [2], and Novel hybrid DL model based on CNN [10]**

| Network samples | F-measure (%) | | | |
| --- | --- | --- | --- | --- |
|  | GM-FVRL | IPro | MACCA2-RF&RF | Novel hybrid DL model based on CNN |
| 500 | 96.47 | 94.46 | 91.93 | 94.89 |
| 1000 | 95.19 | 94.23 | 89.89 | 93.77 |
| 1500 | 94 | 91.57 | 89.07 | 92.77 |
| 2000 | 93.64 | 90.91 | 88.98 | 91.49 |
| 2500 | 92.74 | 91.08 | 88.92 | 91.67 |
| 3000 | 92.95 | 90.40 | 88.89 | 91.34 |
| 3500 | 93.25 | 91.51 | 86.68 | 92.25 |
| 4000 | 93.34 | 90.72 | 89.77 | 92.09 |
| 4500 | 94.17 | 90.98 | 89.49 | 92.27 |
| 5000 | 94.92 | 91.98 | 90.49 | 93.22 |

11. From where were taken the data used in the graphic representations?

**Response:**

To conduct the experiments, the data (i.e., values) are measured in this paper. The data were taken from tables. According to the table values, the graphic representations are plotted in this paper.

12. Some editing "glitches" need to be corrected.

**Response:**

Correction is made in this paper.

13. Punctuations are used randomly. Insert comma or full stop after each and every equation accordingly.

**Response:**

**Correction is made in this paper.**

14. I think the authors need to emphasize more clearly the contribution of the manuscript from a scientific point of view.

**Response: [**contribution of the manuscript mentioned in section 1]

The contributions of GM-FVRL are listed as follows.

- The proposed GM-FVRL is developed for precise network traffic classification with higher accuracy.
- The Gauss Markov Correlation-based Network Traffic Feature Extraction algorithm is employed in GM-FVRL for choosing the optimal network traffic features via the Gauss Markov function by intelligently adjusting the F1-measure function. In this way, the latency is minimized.
- GM-FVRL architecture saves round trip time and enhances accuracy for separating the data plane and control plane via SDN from the corresponding IoT devices.
- GM-FVRL utilizes a flow-balanced radial-based machine learning method to efficiently attain network traffic classifications through a flow-balanced radial basis function with maximum precision and recall rate.
- Experiments of proposed GM-FVRL and existing methods were conducted. Experimental outcomes demonstrate that GM-FVRL has superior latency and attains enhanced performance and stability in classification accuracy and precision compared to conventional network traffic classification methods.

15. References are not uniformly written. In some references the volume, or the issue, or the pages are not specified.

**Response:**

**Volume, or issue, or pages added in references.**

16. Also, I think, the authors must strengthen the References section with some articles that use some similar techniques, to make the techniques used more plausible, for instance: The Effects of Fractional Time Derivatives in Porothermoelastic Materials Using Finite Element Method, Mathematics, 9(14) (2021), Art. No. 1606; On mixed problem in thermos-elasticity of type III for Cosserat media, Journal of Taibah University for Science, 16(1) (2022), 1264–1274.

**Response: [Above references added in section 2]**

The finite element method was introduced in [20] for porothermoelastic media. Temperature increments and stress were discussed by using fractional time derivatives. Linear theory was introduced in [21] for the thermoelasticity of type III for Cosserat media. Uniqueness and continuous dependence were recognized with the logarithmic convexity method.

[20] Marin Marin, Aatef Hobiny and Ibrahim Abbas, (2021) “The Effects of Fractional Time Derivatives in Porothermoelastic Materials Using Finite Element Method”, Mathematics, MDPI, Volume 9, Issue 14, Pages 1-14.

[21] Marin, Marin ,Seadawy, Aly,Vlase, Sorin ,[Chirila, Adina](https://ui.adsabs.harvard.edu/search/q=author:%22Chirila%2C+Adina%22&sort=date%20desc,%20bibcode%20desc), (2022) “On mixed problem in thermos-elasticity of type III for Cosserat media, Journal of Taibah University for Science”, Voulme 16, Issue 1, Pages 1264–1274

If the authors take into account all these corrections, then this manuscript deserves to be published.

**Reviewer #2:**  **Correction is made in green color**

1. In abstract, the existing approach helps to reduce accuracy. Is it meaningful the word "helps"?

**Response:**

The existing approach reduces the accuracy and improves latency.

2. Justify the need of two different parameters duration_sec and duration_nsec as in Table 2.

**Response: [Correction is made in table 2]**

duration_sec, (dur) which is the duration in seconds (dur_sec)

duration_nsec, which is the duration in nanoseconds (dur_nsec)

3. What is the impact for increasing the number of traffic samples to compute precision as in Table 4? There is no linear growth.

**Response:**

To check scalability, the precision is measured with varying number of traffic samples. From the above figure, the precision is neither found to be increasing nor found to be decreasing with the increase in the network traffic samples.

**Precision:**

$$P=\frac{TP}{TP+FP}*100$$

In first iteration, 500 network traffic samples is considered and the precision is measured for proposed GM-FVRL and Existing IPro [1], MACCA2-RF&RF [2], and Novel hybrid DL model based on CNN [10].

$$P \left( GM-FVRL \right)=\frac{480}{480+20}*100=96\%$$

$$P \left( \mathrm{GIPro} \right)=\frac{470}{470+30}*100=94\%$$

$$P \left( MACCA2-RF\&RF \right)=\frac{450}{450+50}*100=90\%$$

$$P \left( MACCA2-RF\&RF \right)=\frac{473}{473+27}*100=94.55\%$$

In second iteration, 1000 network traffic samples is considered and the precision is measured for proposed GM-FVRL and Existing IPro [1], MACCA2-RF&RF [2], and Novel hybrid DL model based on CNN [10].

$$P \left( GM-FVRL \right)=\frac{953}{953+47}*100=95.25\%$$

$$P \left( \mathrm{GIPro} \right)=\frac{932}{932+68}*100=93.15\%$$

$$P \left( MACCA2-RF\&RF \right)=\frac{895}{895+105}*100=89.55\%$$

$$P \left( MACCA2-RF\&RF \right)=\frac{940}{940+60}*100=94\%$$

4. In figure 2, there is some hidden content in "features"

**Response: [23 features added in above equation 1]**

Total 23 features are obtainable in the data set. Extracted features comprise Switch-id, Packet_count, byte_count, duration_sec, duration_nsec, tot_dur, Source IP, Destination IP, Port number, tx_bytes, rx_bytes, flows, dt, pktperflow, byteperflow, pktrate, packetins, pairflow, tx_kbps, rx_kbps, tot_kbps, Port Bandwidth and label.

5. No clarity in Figure 2, no content in ellipse symbol and no proper interconnection

**Response:**

**Correction is made in Figure 2.**

6. Highlight the causes of occurring network traffic.

**Response: [Correction is made in introduction]**

Network congestion occurs when the traffic flowing during a network exceeds its maximum capacity. In several cases, [congestion](https://www.geeksforgeeks.org/congestion-control-in-computer-networks/) is issue with the network caused owing to a sudden upsurge of traffic. But, the network is repeatedly congested, indicating a deeper problem. The **network congestion can cause packet loss, unneeded traffic and increased latency.**

7. Overall organization of the paper is quite novelty and noteworthy.

**Response: [Correction is made in section 1]**

The novelty and contributions of GM-FVRL are listed as follows.

- The proposed GM-FVRL is developed for precise network traffic classification with higher accuracy.
- The Gauss Markov Correlation-based Network Traffic Feature Extraction algorithm is employed in GM-FVRL for choosing the optimal network traffic features via the Gauss Markov function by intelligently adjusting the F1-measure function. In this way, the latency is minimized.
- GM-FVRL architecture saves round trip time and enhances accuracy for separating the data plane and control plane via SDN from the corresponding IoT devices.
- GM-FVRL utilizes a flow-balanced radial-based machine learning method to efficiently attain network traffic classifications through a flow-balanced radial basis function with maximum precision and recall rate.
